# A high-resolution *in vivo* magnetic resonance imaging atlas of the human hypothalamic region

**DOI:** 10.1038/s41597-020-00644-6

**Published:** 2020-09-15

**Authors:** Clemens Neudorfer, Jürgen Germann, Gavin J. B. Elias, Robert Gramer, Alexandre Boutet, Andres M. Lozano

**Affiliations:** Division of Neurosurgery, Department of Surgery, Toronto Western Hospital, University of Toronto, Toronto, Canada

**Keywords:** Brain imaging, Magnetic resonance imaging, Brain, Neuroendocrine diseases

## Abstract

The study of the hypothalamus and its topological changes provides valuable insights into underlying physiological and pathological processes. Owing to technological limitations, however, *in vivo* atlases detailing hypothalamic anatomy are currently lacking in the literature. In this work we aim to overcome this shortcoming by generating a high-resolution *in vivo* anatomical atlas of the human hypothalamic region. A minimum deformation averaging (MDA) pipeline was employed to produce a normalized, high-resolution template from multimodal magnetic resonance imaging (MRI) datasets. This template was used to delineate hypothalamic (n = 13) and extrahypothalamic (n = 12) gray and white matter structures. The reliability of the atlas was evaluated as a measure for voxel-wise volume overlap among raters. Clinical application was demonstrated by superimposing the atlas into datasets of patients diagnosed with a hypothalamic lesion (n = 1) or undergoing hypothalamic (n = 1) and forniceal (n = 1) deep brain stimulation (DBS). The present template serves as a substrate for segmentation of brain structures, specifically those featuring low contrast. Conversely, the segmented hypothalamic atlas may inform DBS programming procedures and may be employed in volumetric studies.

## Abstract (summary)

**Table:** 

Measurement(s)	hypothalamus • information acquisition • gray matter of diencephalon • diencephalic white matter • sexual dimorphism • brain volume measurement • brain segmentation • neuroantomical mapping
Technology Type(s)	Magnetic Resonance Imaging • digital curation
Sample Characteristic - Organism	Homo sapiens

Machine-accessible metadata file describing the reported data: 10.6084/m9.figshare.12888560

## Background & Summary

The hypothalamus is an intricate neuroanatomical structure that occupies 0.3% of the adult human brain volume^1^. Despite its comparatively small size it plays a crucial role in the homeostasis of neuroendocrine, behavioral, and autonomic processes essential to life^2^. Through a vast array of neural connections, it receives input from both spinal cord and brainstem nuclei as well as corticolimbic structures. This information is integrated within dedicated hypothalamic nuclei, altered through secondary and tertiary hypothalamic relay stations and then conveyed to autonomic and limbic control centers where the appropriate physiological response is orchestrated across organ systems^3^. Apart from axonal projections, the hypothalamus further shapes the physiological response via engagement of the pituitary gland and release of hormones into the bloodstream^4^.

Owing to its vital role, the hypothalamus is of great clinical and scientific interest. It has been implicated in a wide range of neurological, endocrinological and psychiatric diseases, is a common drug target, and finds surgical exposure in neurooncology and functional neurosurgery^5^. To understand the causal underpinnings of hypothalamic dysfunction, an extensive body of research is dedicated towards establishing the structural and functional relationships within the hypothalamus proper as well as the local and global structural connectivity wherein its nuclei are embedded^6–8^. In the field of neuroimaging, this effort is primarily directed towards volumetric and topological analyses in healthy and diseased states^9–11^. Conversely, probing of the hypothalamus by means of electrical stimulation and lesioning provides insights into the functional role of individual nuclei^12–15^. The accurate delineation and targeting of the hypothalamus, however, has conventionally been associated with technological and methodological challenges, namely the lack of structural detail and contrast on routinely acquired magnetic resonance imaging (MRI) scans.

To date, an abundance of subcortical atlases has been generated detailing structures commonly targeted during functional neurosurgical procedures such as deep brain stimulation (DBS) surgery. These atlases feature structures such as the thalamus^16–18^, subthalamic nucleus (STN)^19,20^, and globus pallidus (GP)^21^. By contrast, the hypothalamus and its surrounding landmarks have remained largely underrepresented. Several groups have accurately delineated the hypothalamus on MRI sequences using manual and semiautomatic segmentation methods^8,11,22,23^. The majority of studies, however, resorted to identifying the hypothalamus as a single structure. While this approach may suffice for the measurement of overall hypothalamic volumes, the lack of morphological detail prevents more sophisticated analyses at the nuclear level. For clinical applications (e.g., DBS lead localization, stimulation titration, or evaluation of tumor infiltration) and research purposes (e.g. volumetric comparisons of nuclei in physiological and pathological brain states), however, a higher degree of detail is desirable.

In the present study we aimed to overcome this long-standing hurdle by adopting a “big data” approach to the identification of hypothalamic nuclei. We generated a high-resolution, high-contrast template comprised of a total of 990 brain scans that allowed us to identify and segment individual hypothalamic nuclei and their neighboring structures. We investigated the clinical applicability of the generated atlas to individual patients and demonstrate its potential utility for electrode localization as well as volumetric analyses.

## Methods

### Data acquisition

The source data for the atlas were drawn from structural magnetic resonance image (MRI) datasets from the Human Connectome Project (HCP) S1200 subject release (https://www.humanconnectome.org/study/hcp-young-adult/document/1200-subjects-data-release). Scans were obtained following 3.0 Tesla MRI using the following acquisition parameters: (1) T1- MPRAGE: TR = 2400 ms; TE = 2.14 ms; TI = 1000 ms; Flip angle = 8 deg; FoV = 224 × 224 mm; voxel size = 0.7 mm isotropic; (2) T2-SPACE: TR = 3200 ms; TE = 565 ms; Flip angle = variable; FoV = 224 × 224 mm; voxel size = 0.7 mm isotropic. The Minimal Preprocessing Pipeline (MPP) had been applied to all structural imaging files and included spatial/artefact distortion removal, cross-modal registration, surface generation, and alignment to standard space^24^. During preprocessing, T1- and T2-weighted scans of young (age range: 22–35 years), healthy individuals were selected; In total, 1000 datasets were downloaded from the S1200 subject release; the selection of datasets did not follow a systematic approach. Incomplete and corrupted files that were deemed inappropriate upon visual inspection were subsequently excluded from further analysis (n = 10). If a patient dataset comprised multiple versions of the same modality all versions were inspected visually and the most suitable one was selected based on image quality. The final data set yielded 990 individual scans that were subsequently used for further processing.

### Image processing and template generation

**Fig. 1:**
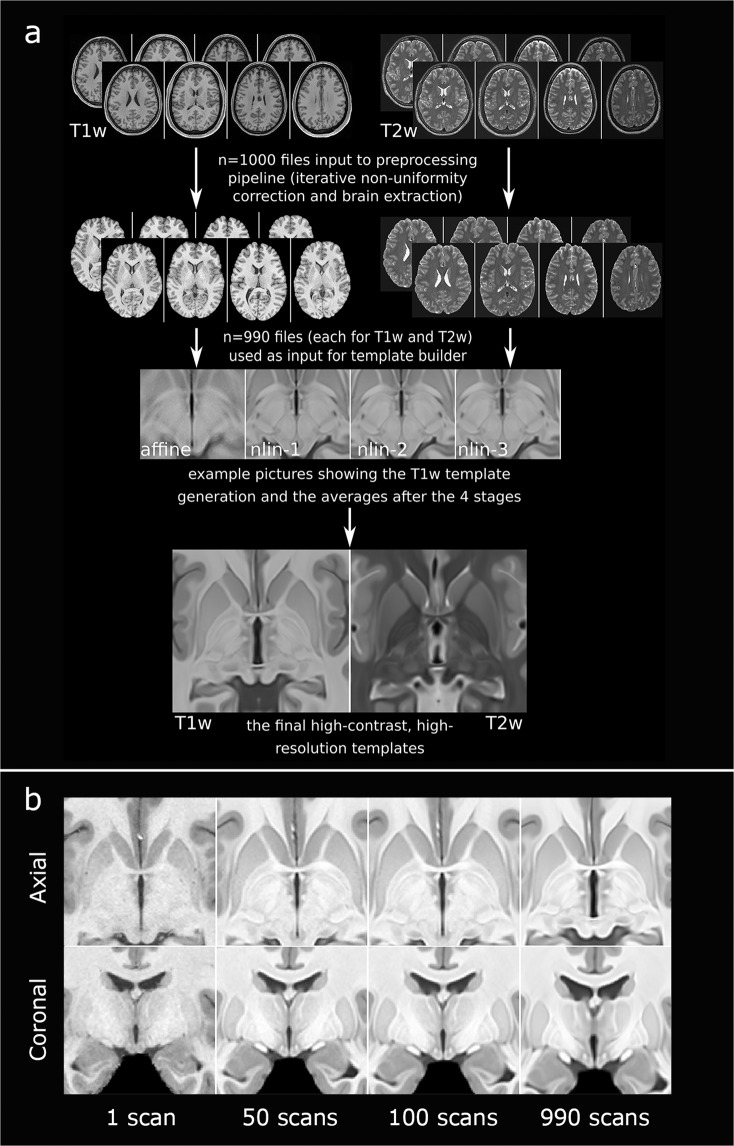
Workflow for the generation of the multimodal high-resolution MRI template. (**a**) 1000 minimally preprocessed T1-weighted (T1w) and T2-weighted (T2w) MRI scans were drawn from the HCP S1200 subject release. Incomplete and corrupted imaging data was excluded during preprocessing yielding 990 scans that were ultimately selected for template generation. Following preprocessing, the antsMultivariateTemplate builder was applied to the scans to perform one round of affine and three rounds of iterative non-linear registration. This process yielded a high-contrast, high-resolution MRI template of great anatomical detail (voxel size: 0.25 × 0.25 × 0.25 mm). Based on this dataset, a second, template was generated (voxel size: 0.5 × 0.5 × 0.5 mm). (**b**) Image resolution and contrast across differently sized populations. Representative axial and coronal sections of processed T1w (top) and T2w (bottom) MRI scans featuring the increase in tissue contrast during registration of differently sized populations (one, 50, 100 and 990 subjects). The complete MDA pipeline consisting of one round of affine and three rounds of non-linear registration had been applied to all populations. The resulting high tissue contrast in the final templates facilitated hypothalamic segmentation and allowed visualization of tissue boundaries that would have otherwise not been delimitable. MDA, minimum deformation averaging.

The herein employed technique to generate multi-sequence average templates from 990 individual MRI scans is referred to as Minimum deformation averaging (MDA)^25^. MDA exploits the information contained within inter-individual variations to generate an unbiased, high-resolution, high-contrast population average. Through iterative model building, single-subject data is repeatedly aligned to capture the average morphology of the population used in model generation, yielding a final template of superior imaging quality. The MDA pipeline applied in the generation of our multimodal templates is featured in Fig. 1 and described as follows:

For each subject, MRI datasets were initialized using the minc-bpipe-library pipeline^26^. An iterative, non-uniform correction of field inhomogeneities was initially performed using the ANTs N4 correction tool^26,27^. The BEaST brain extraction software was then employed to process the brain masks (BM) and skull strip both T1- and T2-weighted images^28^. Finally, to achieve a standardized output, the image axis was aligned to MNI152 NLIN 2009b space and the field-of-view was cropped.

Following preprocessing, the antsMultivariateTemplate builder (https://github.com/ANTsX/ANTs/blob/master/Scripts/antsMultivariateTemplateConstruction2.sh) was applied to the imaging dataset to generate the group averages. This entailed the use of a hierarchical image registration algorithm that applied a total of four rounds of registration to the imaging data-set in a stepwise manner. All steps were performed independently for the T1- and T2-weighted scans. During the first step, a MNI152 NLIN 2009b template (voxel size: 0.25 × 0.25 × 0.25 mm) was used as the initial target to perform an affine registration step scaling the 990 individual scans to MNI space^29^. In the same instance, the individual subject scans were upsampled to the target resolution using B-spline; the spatial information contained within the scans, however, remained unchanged. Subsequently, three rounds of non-linear registration were performed. During each round of non-linear registration, an average was generated from the brains registered in the previous round and that average was then used as the target for the subsequent registration. Each iteration consequently produced a more detailed average. The resolution and contrast of each subsequent average were qualitatively superior to the previous step (Fig. 1). The final template yielded a high-resolution, high-contrast dataset of enhanced anatomical detail (voxel size: 0.25 × 0.25 × 0.25 mm). Although the spatial resolution of the original subject scans (0.7mm^3^) would have potentially have allowed us to generate a final template of even higher resolution, a voxel size of 0.25 mm isotropic resolution was deemed sufficient as further increases in resolution would have yielded considerably longer processing times and higher memory demand. In addition, voxel resolutions beyond 0.25 × 0.25 × 0.25 mm would find little application in existing MRI techniques. A second template (voxel size: 0.5 × 0.5 × 0.5 mm) was generated by downsampling the high-resolution template. This template may be used on computers with reduced processing power or for segmentations requiring less anatomical detail.

The final step of template construction involved the transformation of T1- and T2-weighted templates into MNI space. To this end, the final T1-weighted dataset was non-linearly aligned to an MNI152 NLIN 2009b template using antsRegistration. The same software was then employed to non-linearly register the T2-weighted template to the T1-weighted template.

### Manual segmentation of hypothalamic nuclei and atlas generation

**Fig. 2:**
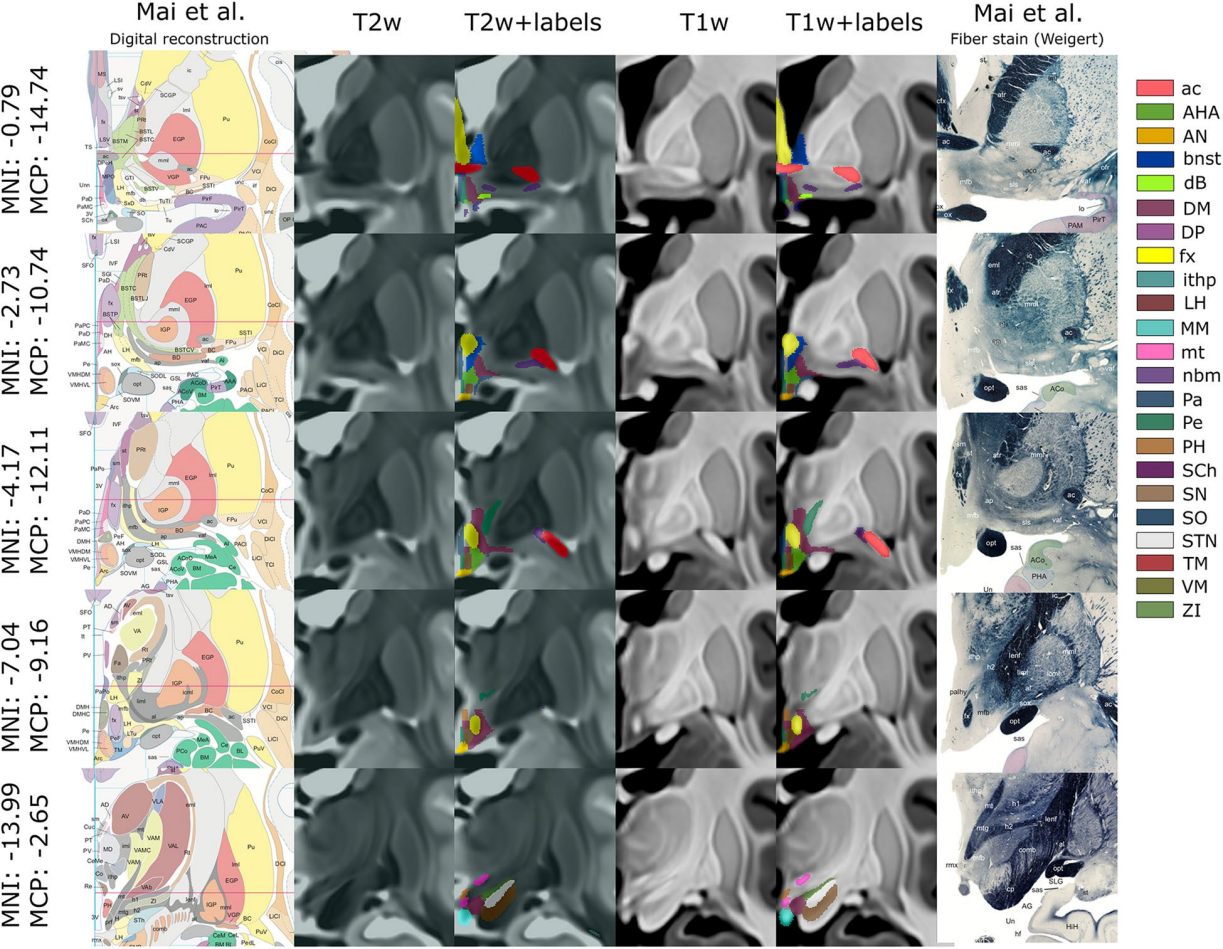
2D representation of the hypothalamic atlas. Manually segmented hypothalamic and extrahypothalamic labels are overlaid on representative coronal T2-weighted (T2W) (two center left columns) and T1-weighted (T1W) (two center right columns) sections. Montreal Neurological Institute (MNI152 NLIN 2009b) coordinates and the distance from the midcommissural point (MCP) are shown. Reconstructions (left column) and histologic sections (right column) from the Atlas of the human brain were used to facilitate the delineation of hypothalamic and surrounding structures. ac, anterior commissure; AHA, anterior hypothalamic area; AN, arcuate nucleus; BNST, bed nucleus of stria terminalis; dB, diagonal band of Broca; DM, dorsomedial hypothalamic nucleus; DP, dorsal periventricular nucleus; fx, fornix; ithp, inferior thalamic peduncle; LH, lateral hypothalamus; MM, mammillary bodies; mt, mammillothalamic tract; nbm, nucleus basalis of Meynert; PA, paraventricular nucleus; PE, periventricular nucleus; PH, posterior hypothalamus; RN, red nucleus; SCh, suprachiasmatic nucleus; SN, substantia nigra; SO, supraoptic nucleus; STN, subthalamic nucleus; TM, tuberomammillary nucleus; VM, ventromedial nucleus; ZI, zona incerta. (Reproduced with permission from Mai JK, Paxinos G, Voss T (2016): Atlas of the Human Brain, 4^th^ ed. San Diego: Elsevier Academic Press.

**Fig. 3:**
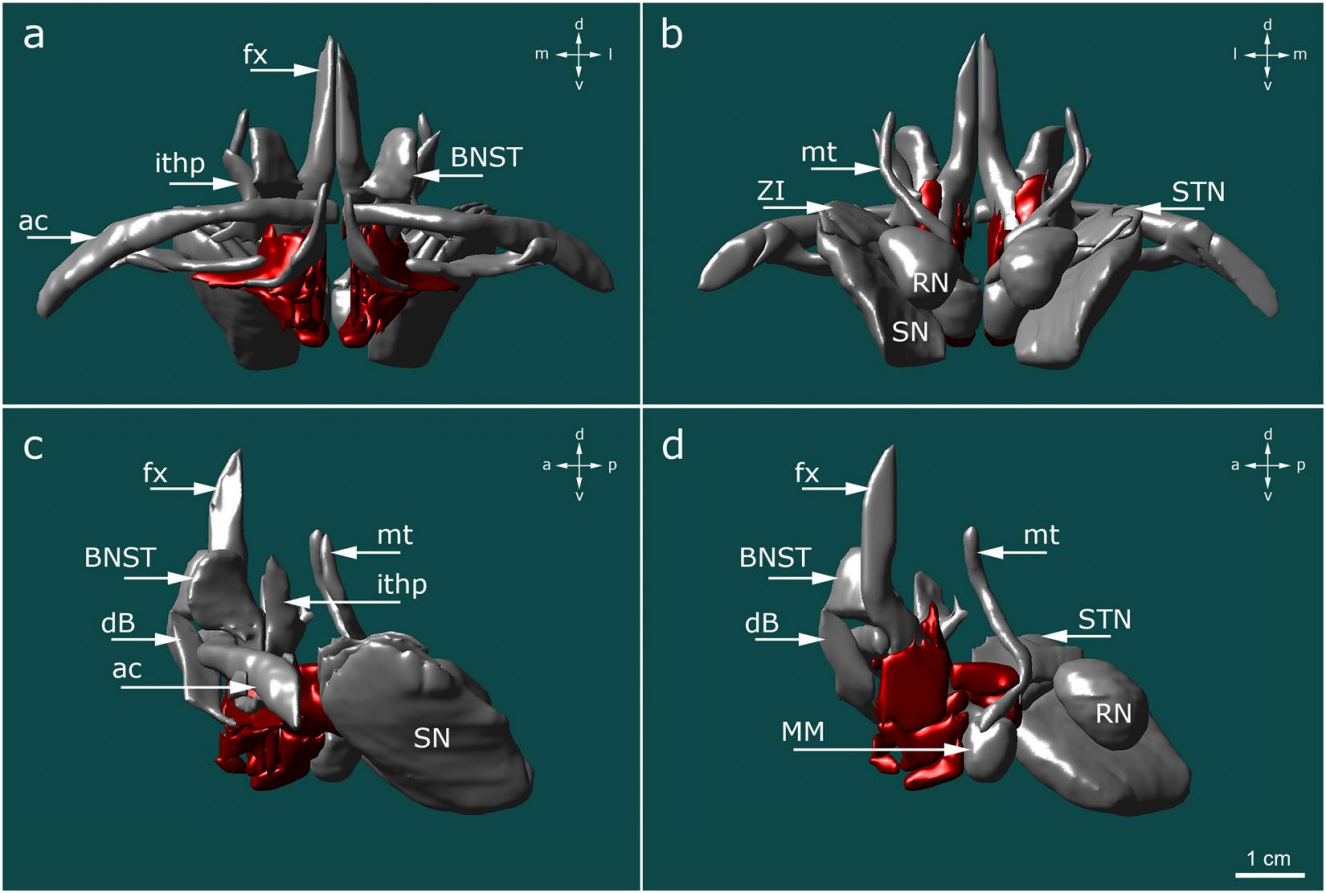
3D reconstruction featuring the topographic relationships between the hypothalamus and surrounding gray and white matter structures. (**a**) frontal view; (**b**) occipital view; (**c**) sagittal view of the hypothalamus (red) and surrounding structures (gray); (**d**) sagittal view of the medial surface of the right hypothalamus (red) and surrounding structures (gray). The anterior confines of the hypothalamus are comprised of the diagonal band of Broca (dB) and the anterior commissure (ac). The latter is directly neighbored by the bed nucleus of the stria terminalis (BNST) as well as the fornix (fx), which penetrates through the hypothalamus and terminates in the mammillary bodies (MM). Along with the mammillothalamic tract (mt), the fornix and mammillary bodies form part of the Papez circuit. The lateral boundary of the hypothalamus is formed by the subthalamic nucleus (STN), substantia nigra (SN), zona incerta (ZI), and red nucleus (RN). The lateral border is extended anteriorly by the inferior thalamic peduncle (ithp).

**Fig. 4:**
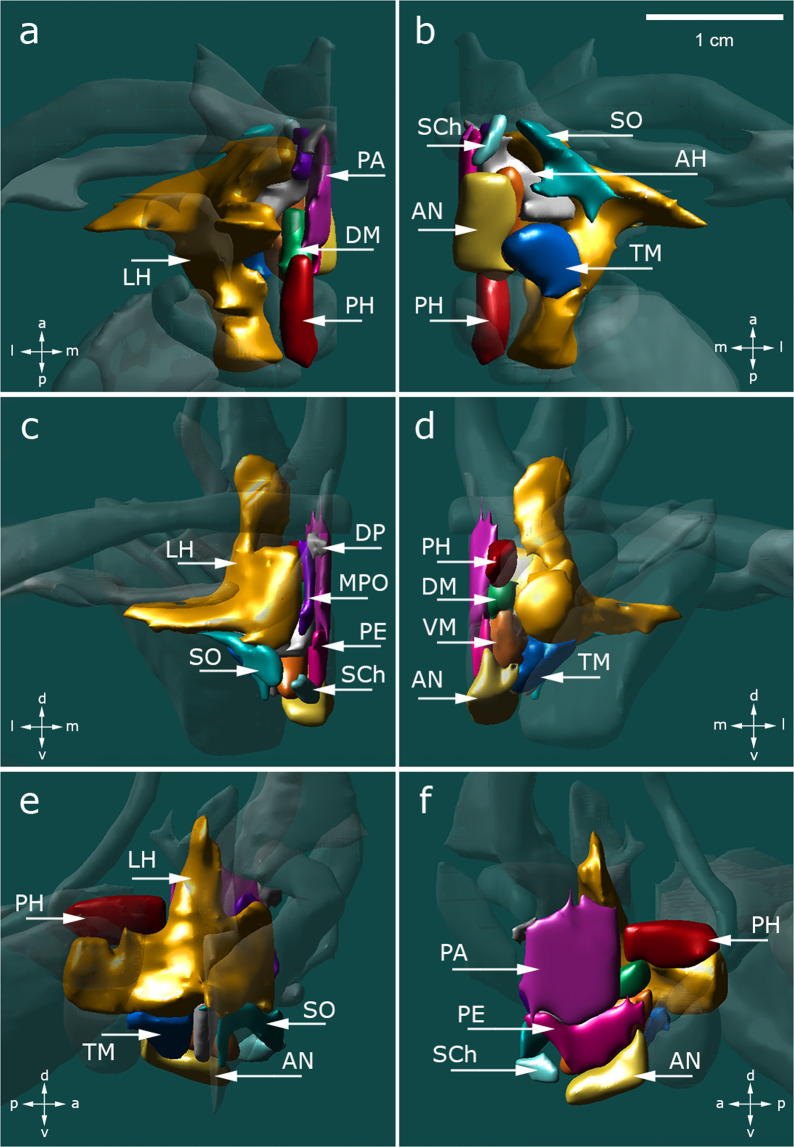
3D reconstruction of hypothalamic nuclei and their neuroanatomical relationships. (**a**) top view; (**b**) bottom view; (**c**) frontal view; (**d**) occipital view; (**e**) sagittal view depicting the outer surface of hypothalamic nuclei; (**f**) sagittal view depicting the inner surface of hypothalamic nuclei. Surrounding structures unrelated to the hypothalamus proper are transparent. AH, anterior hypothalamic area; AN, arcuate nucleus; DP, dorsal periventricular nucleus; DM, dorsomedial hypothalamic nucleus; LH, lateral hypothalamus; MPO, medial preoptic nucleus; PA, paraventricular nucleus; PE, periventricular nucleus; PH, posterior hypothalamus; SCh, suprachiasmatic nucleus; SO, supraoptic nucleus; TM, tuberomammillary nucleus; VM, ventromedial nucleus.

The hypothalamic atlas was generated by two raters (CN and JG) who manually labeled diencephalic and mesencephalic structures bilaterally using Display (Montreal Neurological Institute; http://www.bic.mni.mcgill.ca/software/Display/Display.html) (Fig. 2). To allow fine-grained labeling at great anatomical detail, segmentation was performed using the high-resolution, high-contrast template of enhanced anatomical detail (voxel size: 0.25 × 0.25 × 0.25 mm). The primary reference for the delineation of hypothalamic nuclei and surrounding structures was the Atlas of the human brain, fourth edition^30^. The Allen Brain Atlas (http://www.brain-map.org) served as a secondary reference^31^. Volumes were defined on T1-weighted (T1w) and T2-weighted (T2w) images. To facilitate the delineation of hypothalamic nuclei, structures with explicit tissue boundaries (i.e., clear contrast between regions) were labeled first. These included, among others, the anterior commissure, diagonal band of Broca, fornix, mammillothalamic tract, mammillary bodies, subthalamic nucleus, and substantia nigra (Fig. 3). White matter structures were included if they featured high-contrast borders (e.g. in close proximity to gray matter structures or cerebrospinal fluid). The borders of these anatomical landmarks were used to delineate implicit brain regions of low tissue contrast (Fig. 4). Structures that featured low contrast (e.g. medial forebrain bundle (mfb), fields of Forel, mammillotegmental tract) were not segmented. Of note, targeting of mfb has thus far relied on diffusion tensor imaging and tractographic identification; delineation of mfb based on structural MRI only is not possible^32,33^. Following labeling the dice similarity coefficient (DSC) was calculated for each label to determine the extent of inter-rater agreement. Labels associated with a DSC < 0.35 were reevaluated jointly and amended by both raters such that neuroanatomical differences were reduced to a minimum^23,34,35^. For the generation of the final diencephalic Atlas an OR logical was employed i.e. the label that was overall deemed more accurate was implemented in the final Atlas build.

## Data Records

### Data records as a contribution

**Fig. 5:**
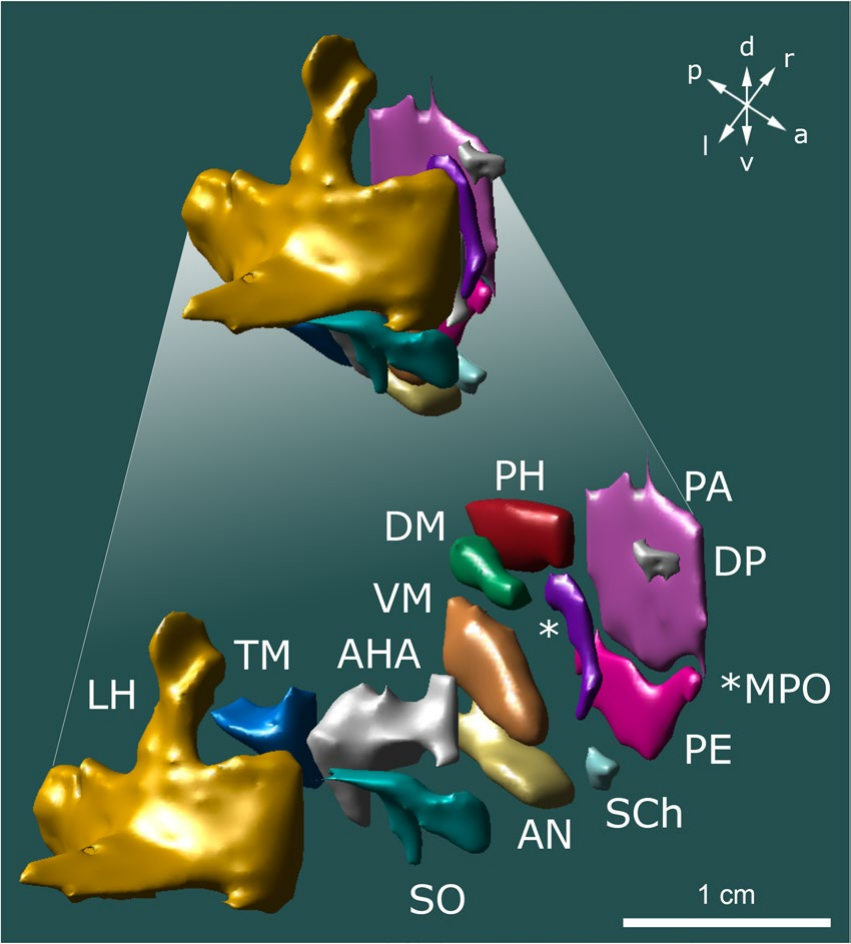
Spatial relationship between individual hypothalamic nuclei. Hypothalamic nuclei are depicted in a consolidated (top) and an expanded view (bottom) revealing the intrahypothalamic relationships across nuclei. AHA, anterior hypothalamic area; AN, arcuate nucleus; DP, dorsal periventricular nucleus; DM, dorsomedial hypothalamic nucleus; LH, lateral hypothalamus; MPO, medial preoptic nucleus; PA, paraventricular nucleus; PE, periventricular nucleus; PH, posterior hypothalamus; SCh, suprachiasmatic nucleus; SO, supraoptic nucleus; TM, tuberomammillary nucleus; VM, ventromedial nucleus.

The contribution of the presented work are two data records featuring the minimal deformation, age- and population-averaged high-resolution templates and segmentations of the hypothalamic region. The high-resolution template comprises T1- and T2- weighted image files given in NIfTI-1 format. Both scans are available in ultra-high (voxel size: 0.25 × 0.25 × 0.25 mm) and high (voxel size: 0.5 × 0.5 × 0.5 mm) resolution. The second data record features the atlas of the hypothalamic region and contains a total of 25 subcortical gray and white matter regions per hemisphere. 13 of the 25 anatomical labels constitute hypothalamic nuclei (Fig. 5), while the remaining labels comprise adjacent structures such as fornix, inferior thalamic peduncle, nucleus basalis of Meynert, and bed nucleus of the stria terminalis. These structures were segmented owing to their clinical importance and employment as targets for DBS surgery. The high contrast and resolution of the template greatly facilitated hypothalamic segmentation and allowed visualization of tissue boundaries that would have otherwise not been delimitable (Fig. 2). Diencephalic structures are featured as full volume segmentations in 0.25- and 0.5-millimeter isotropic resolution. Furthermore, an archive featuring each respective structure in 0.25 mm resolution is supplied. A detailed description of the labeling criteria used to identify individual hypothalamic nuclei can be obtained from Sup. Table 1. Finally, we provide an archive of the three example datasets that were used for validation of the hypothalamic atlas. It features the volumes of tissue activated (VTAs) of two DBS patients as well as the delineation of a solitary metastasis in MNI152 NLIN 2009b space.

The data records are supplied with a lookup table (stored as.csv file), comprising diencephalic structures featuring information about the respective label code, name, and laterality. All files can be opened with standard visualization software such as fsleyes, Display, and freeview. In addition, the data contribution is supplied by volumetric measurements of mean hypothalamic volumes stratified by gender, laterality, and major hypothalamic subdivisions.

The data records derived from this work can be obtained through Zenodo^36^.

### Original datasets used

The original data used for template construction were provided by the Human Connectome Project, WU-Minn Consortium (Principal Investigators: David Van Essen and Kamil Ugurbil; 1U54MH091657) funded by the 16 NIH Institutes and Centers that support the NIH Blueprint for Neuroscience Research; and by the McDonnell Center for Systems Neuroscience at Washington University. It is available at https://www.humanconnectome.org/study/hcp-young-adult/document/1200-subjects-data-release.

## Technical Validation

### Dice similarity coefficients

To determine the reliability of the segmented parcels, the Dice similarity coefficient (DSC) was calculated as a measure of voxel-wise volume overlap across and within raters. To calculate the index, we used the standard definition of DSC, that is specified as the ratio of the intersection volume of two labels, X and Y, to the mean volume of the respective labels^37^.

$$DSC=\frac{2(\left|X\cap Y\right|)}{\left|X\right|+\left|Y\right|}$$

A threshold value of 0.35 was defined and only DSCs above this value were deemed appropriate^23,34,35^. Structures where such a rater agreement could not be achieved were excluded from the final version of the atlas.

**Fig. 6:**
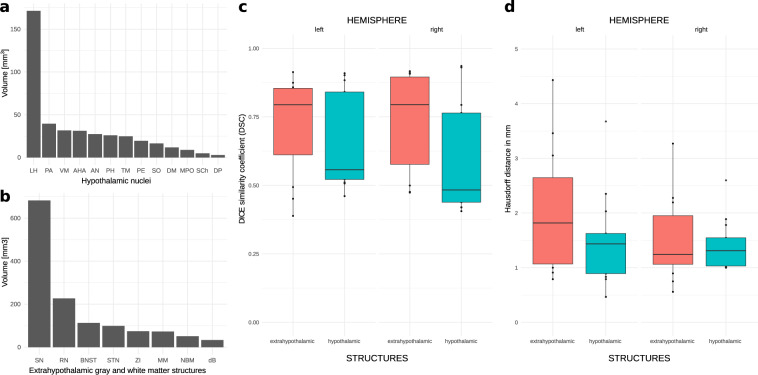
Volume estimates of hypothalamic nuclei and evaluation of interrater agreement during manual segmentation. Bar graphs displaying volume estimates for manually segmented hypothalamic nuclei and (**a**) surrounding gray and white matter structures (**b**). Volumes of structures that were only partially segmented (e.g. anterior commissure and fornix) are not reported. (**c**) Spatial overlap between segmented structures and the extent of voxel-wise agreement across raters were calculated using the dice similarity coefficient (DSC) score and (**d**) Hausdorff distances. Box plots feature the calculated values for hypothalamic nuclei and surrounding (extrahypothalamic) structures within each respective hemisphere. Note that extrahypothalamic structures featured an overall stronger tissue contrast on T1w and T2w images, which ultimately yielded a higher interrater agreement as indicated by a greater DSC score. AH, anterior hypothalamic area; AN, arcuate nucleus; BNST, bed nucleus of stria terminalis; dB, diagonal band of Broca; DP, dorsal periventricular nucleus; DM, dorsomedial hypothalamic nucleus; LH, lateral hypothalamus; MM, mammillary bodies; MPO, medial preoptic nucleus; NBM, nucleus basalis of Meynert; PA, paraventricular nucleus; PE, periventricular nucleus; PH, posterior hypothalamus; RN, red nucleus; SCh, suprachiasmatic nucleus; SN, substantia nigra; SO, supraoptic nucleus; STN, subthalamic nucleus; TM, tuberomammillary nucleus; VM, ventromedial nucleus; ZI, zona incerta.

Voxel-wise calculation of DSC scores was performed for hypothalamic nuclei and surrounding gray and white matter structures (Fig. 6c). Evaluation across raters revealed greater overlaps in structures with explicit tissue boundaries such as paraventricular nucleus and fornix. By contrast, structures of low contrast including dorsomedial and suprachiasmatic nucleus featured larger deviations. Mean DSC scores, however, were within acceptable margins of error for both hypothalamic nuclei (mean ± SD = 0.63 ± 0.19, range: 0.41–0.94) and surrounding structures (mean ± SD = 0.75 ± 0.18, range: 0.39–0.86) and conformed to values typically reported in the literature^23,34^. Evaluation of within-rater variability on a subset of labels yielded similar values (mean ± SD = 0.66 ± 0.12, range: 0.47–0.82) suggesting consistency of segmentation across multiple instances (Supplementary Table 1). An itemized description of DSCs can be obtained from Supplementary Table 2.

### Hausdorff distances

To calculate the non-directed Hausdorff distance (HD) between two labeled regions, the minimum Euclidian distance from each voxel in one label X to any voxel in a corresponding label Y was measured^38^. The maximum of the resulting minimum distances was then calculated using the following formula:

$$HD\left(X,Y\right)={\rm{\max }}\{hd\left(X,Y\right),hd(Y,X)\}$$

where hd(X,Y) and hd(Y,X) constitute the forward and backward Hausdorff distances of X to Y that yield the non-directed HD. Evaluation of HDs across raters revealed a mean distance of 1.44 ± 0.67 mm (range: 0.47–3.67 mm) for hypothalamic nuclei and 1.79 ± 1.00 mm (range: 0.55–4.43 mm) for extrahypothalamic nuclei, respectively (Fig. 6d). Assessment of within-rater variability yielded a mean HD of 1.66 ± 0.77 mm (range: 1.00–3.32)(Supplementary Table 1). For an itemized description of HDs refer to Supplementary Table 2.

### Applicability to clinical and research settings

**Fig. 7:**
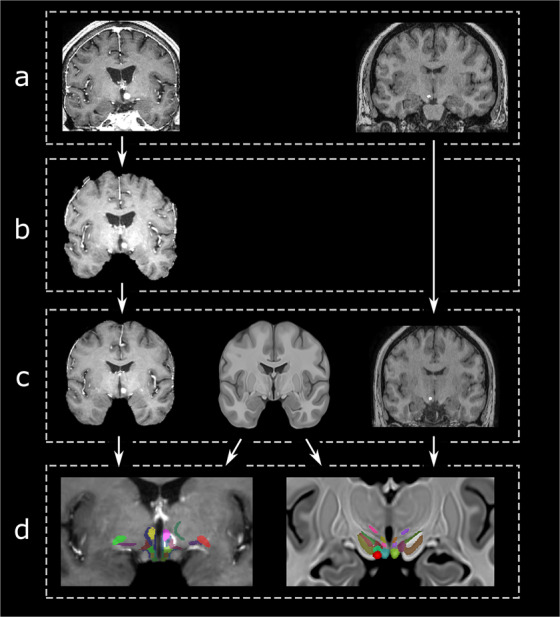
Workflow for the clinical application of the hypothalamic atlas in a patient suffering from a hypothalamic lesion (solitary cerebral metastasis, left) and for postoperative VTA modeling in a patient receiving hypothalamic DBS (right) (**a**) The patients’ native brain scans were skull stripped (**b**) and transformed into template space (**c**). This was achieved via affine and non-linear registration of the preprocessed MRI datasets (c, left and right) to the high-resolution template (c, center). (**d**) features the final registration result.

The lack of morphological detail in conventional MRI scans drastically restricts the direct identification of the hypothalamus proper. The availability of a reference atlas, however, could provide valuable anatomical information that might facilitate postoperative delineation of lesions or lead localization, while potentially reducing side effects during electrical stimulation. Thus, to validate the applicability of our atlas in a clinical setting, we applied the atlas to three individual subjects (Fig. 7). Specifically, we investigated the location of the volume of tissue activated (VTA) as an estimation of stimulated tissue and structures in a 77-year-old patient with a two-year history of Alzheimer’s disease who underwent forniceal deep brain stimulation (DBS). DBS was offered to the patient within the context of an ongoing randomized, controlled trial (NCT01608061) and the experimental phenomena observed with stimulation in this patient have been reported previously^39^. In a second patient, VTA estimation was performed to correlate side-effects of acute electrical stimulation with electrode localization in the lateral hypothalamus (LH). The patient is part of an ongoing clinical trial at our center (NCT03650309) and was recruited for DBS in the treatment of morbid obesity (BMI: 42.3). Owing to weight fluctuations and issues with food cravings the patient was offered DBS surgery as a treatment alternative. Finally, we localized a hypothalamic metastasis in a 67-year-old male suffering from recurrent oral cavity cancer. In the course of follow-up, an MRI scan of the brain revealed an incidental hypothalamic lesion in vicinity to the optic chiasm. Clinically, no visual impairment or focal neurological deficits related to the lesion were noted.

In all patients, T1w scans were transformed into template space using an affine registration step followed by non-linear registration (Fig. 7). After visually confirming the quality of registration, the patient data was overlaid onto the atlas. To visualize the volumes of tissue activated (VTAs) for the two DBS patients, stimulation parameters were modeled in Lead-DBS (http://lead-dbs.org/)^40^ and imported into Display for visualization.

**Fig. 8:**
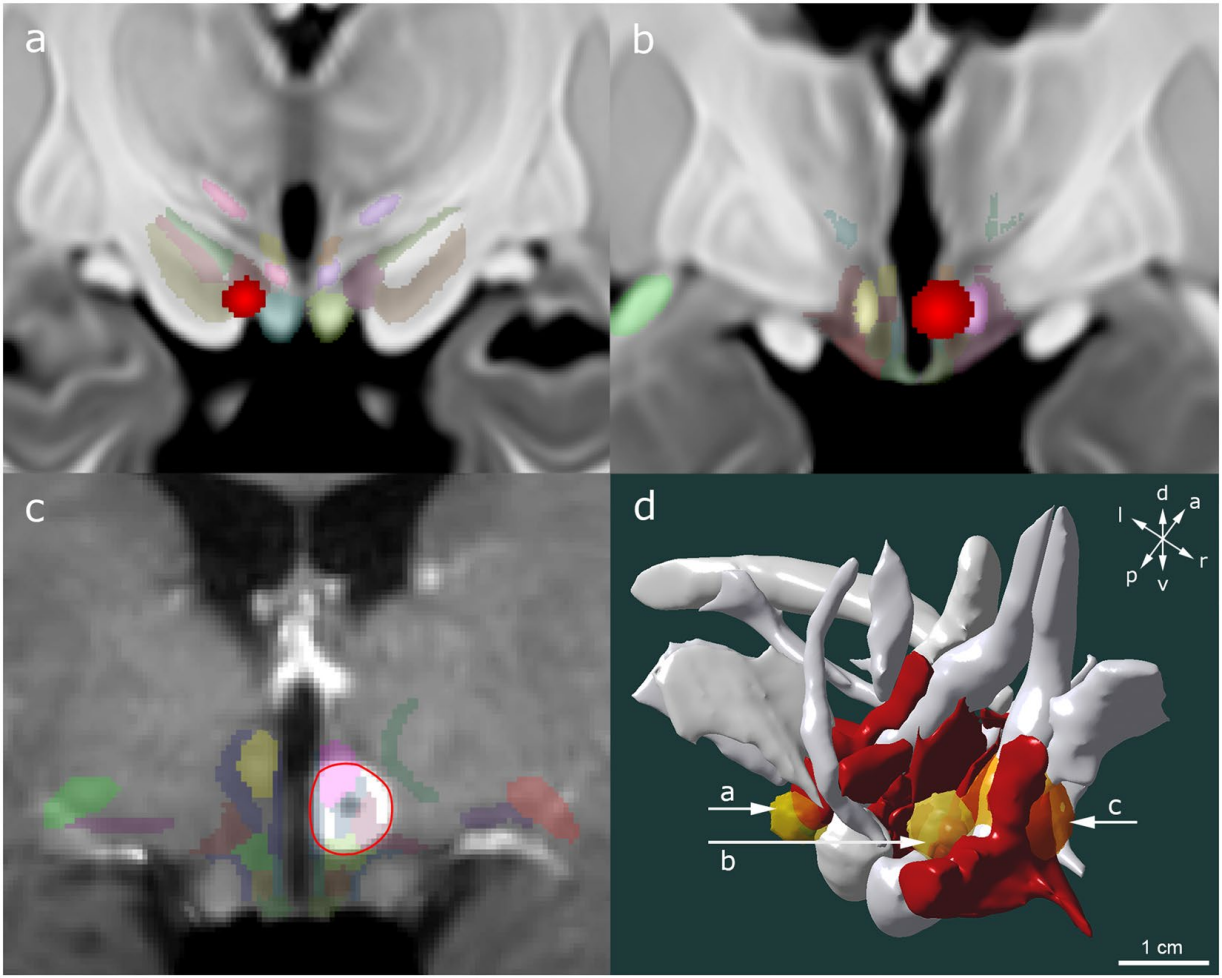
Clinical application of the hypothalamic atlas. Volumes of tissue activated (VTAs, shaded red) of patients receiving (**a**) hypothalamic deep brain stimulation (DBS) in the treatment of morbid obesity and (**b**) forniceal stimulation in Alzheimer’s disease. VTAs were overlaid on representative coronal sections from the T1w high-resolution MRI template. The superimposed hypothalamic atlas depicts the neural substrate encompassed during electrical stimulation. (**c**) An incidental, contrast-enhancing solitary hypothalamic metastasis (red circle) in a patient who had previously been diagnosed with a melanoma. The patient’s T1w MRI scan was registered with the high-resolution template and the atlas superimposed. (**d**) Oblique view of a hypothalamic 3D reconstruction featuring the spatial relationship between VTAs (shades of yellow), metastasis (shades of orange), hypothalamic nuclei (red), and surrounding structures (gray). The posterior hypothalamus (PH) was removed on the right side to allow visualization of VTAs.

Registration of patient T1w scans, VTAs and labels to template space proved effective in all patients as confirmed visually. Figure 8 illustrates the location of the metastasis and VTAs with respect to their surrounding anatomy following image processing and transfer into template space.

Of note, the atlas does not take into account tissue displacement as would be expected during repressive tumor growth (e.g. in cerebral metastases). In clinical settings, however, the atlas may provide valuable insight into the general location of the lesion with respect to the surrounding anatomy and correlation with observed symptoms.

Symptoms and side-effect profiles as reported during stimulation programming correlated with electrode location and the nuclear structures encompassed by the computed VTAs. Acute electrical stimulation of LH in the patient undergoing DBS for surgically refractory morbid obesity yielded autonomic responses such as increased salivation and body warmth using the following stimulation settings: c + , 2-, 90 µs, 130 Hz, 2.0 mA. Gradual current increases to 6.0 mA induced additional side effects such as heat, sweating and dizziness. Symptoms immediately subsided upon stimulation cessation. Modeling of the 2.0 mA VTA revealed confinement of electrical current to the posterior aspect of LH (Fig. 8a)(Supplementary Table 3).

Electrical stimulation of the fornix in the 77-year-old patient with Alzheimer’s disease induced both, acute recollections of memory and autonomic responses (stimulation settings: c + , 2−, 90 µs, 130 Hz, 4.0 V)^39^.Voltage increases up to 8.0 V exacerbated autonomic and associative symptoms, yielding heart rate increases from a baseline-value of 58/min to 74/min and more vivid recollections of memory. VTA computation of the 4.0 V setting revealed encroachment of electric current on the fornix and several surrounding hypothalamic structures, namely the ventromedial and dorsomedial hypothalamic nuclei (Fig. 8b). The latter relay information to brain regions associated with the regulation of stress, temperature, and cardiovascular function (Supplementary Table 3)^41–43^.

### Volumetric analysis

Volumetric analyses of parcellated hypothalamic nuclei have provided valuable insights into structural changes in physiological and pathological brain states^8,44–48^. In the present study, we compiled the overall and gender-specific volumes for individual hypothalamic nuclei and surrounding structures in a young (range: 22–35 years old), healthy population; these may serve as a substrate for future investigations into structural and functional changes in physiological and pathological brain states. Volumes were estimated by individually registering the final atlas segmentations to the 990 transforms that were used during template construction and computing the Jacobian determinant of the transformation in a voxel-wise manner. Gender-matched Jacobian determinants were then calculated as a measure of volume change between the final template and the gender-specific datasets. In addition, hemispheric and gender-specific volumes were normalized by hemispheric and total brain volume, respectively, to account for interindividual variability (Supplementary Tables 4 and 5).

The mean absolute volume (± SD) of the segmented hypothalamus was 832.9 mm^3^ (± 107.59 mm^3^; left: 429.54 mm^3^ ± 55.23 mm^3^; right: 403.39 mm^3^ ± 53.09 mm^3^). Owing to an overall increase in brain volume, the mean volume in the male subgroup (892.5 mm^3^ ± 99.5 mm^3^; left: 459.82 mm^3^ ± 51.55 mm^3^; right: 432.64 mm^3^ ± 48.90 mm^3^) exceeded the values measured in the female subgroup (782.1 mm^3^ ± 86.05 mm^3^; left: 403.67 mm^3^ ± 44.00 mm^3^; right: 378.41 mm^3^ ± 42.78 mm^3^) (Fig. 6). Table 1 displays overall and gender-specific volume measurements for individual nuclei and tracts. Table 2 features left and right differences. Volumes associated with established hypothalamic subdivisions can be obtained from Table 3, whereas normalized volumes can be obtained from Supplementary Tables 4 and 5.

**Table 1:** List of subcortical gray and white matter structures and gender-specific absolute volumes.

	Name	Abbreviation	Mean volume [mm^3^]					
			Overall	SD	Male	SD	Female	SD
Gray matter	Anterior hypothalamic area	AHA	31.2	4.56	33.57	4.28	29.17	3.74
	Arcuate nucleus	AN	27.34	4.07	29.09	3.92	25.83	3.56
	Bed nucleus of the stria terminalis	BNST	112.86	15.63	122.18	13.9	104.91	12.27
	Dorsal periventricular nucleus	DP	2.87	0.53	3.15	0.52	2.63	0.41
	Dorsomedial hypothalamic nucleus	DM	11.84	1.63	12.42	1.65	11.35	1.44
	Lateral hypothalamus	LH	171.53	22.29	184.5	20.45	160.46	17.26
	Mammillary bodies	MM	73.06	11.46	79.94	10.43	67.19	8.7
	Medial preoptic nucleus	MPO	8.84	1.29	9.52	1.21	8.26	1.04
	Nucleus basalis of Meynert	NBM	50.81	8.18	56.03	7.6	46.35	5.63
	Paraventricular nucleus	PA	39.53	5.7	42.07	5.63	37.36	4.79
	Periventricular nucleus	PE	19.52	2.96	20.97	2.87	18.43	2.57
	Posterior hypothalamus	PH	25.93	3.35	27.55	3.23	24.54	2.77
	Red nucleus	RN	226.88	33.68	246.96	30.85	209.73	25.54
	Substantia nigra	SN	682.29	91.86	739.57	81.9	633.38	68.77
	Subthalamic nucleus	STN	98.93	14.67	108.28	13.14	90.94	10.65
	Suprachiasmatic nucleus	SCh	4.94	0.86	5.2	0.87	4.72	0.79
	Supraoptic nucleus	SO	16.46	2.37	17.66	2.29	15.45	1.91
	Tuberomammillary nucleus	TM	24.76	3.56	26.81	3.22	23.02	2.83
	Ventromedial hypothalamus	VM	31.7	4.64	33.89	4.39	29.84	4
	Zona incerta	ZI	74.14	11.11	81.21	10.05	68.11	8
White matter	Anterior commissure	ac	incomplete segmentation					
	Diagonal band of broca	dB	33.42	4.59	36.33	4.04	30.94	3.42
	Fornix	fx	incomplete segmentation					
	Inferior thalamic peduncle	ithp	incomplete segmentation					
	Mammillothalamic tract	mt	incomplete segmentation					

Average and gender-specific volumes of gray and white matter structures were estimated following individual registration of the final atlas segmentations to the 990 transforms obtained from the HCP S1200 subject release. Overall, 25 individual diencephalic and mesencephalic structures were identified. 13 structures constitute hypothalamic nuclei, while the remaining structures surround the hypothalamus and form natural boundaries to the hypothalamus. For an itemized description of gender-specific volumes normalized by total brain volume refer to Supplementary Table 5.

**Table 2:** List of average absolute left and right hemispheric hypothalamic volumes.

	Name	Abbreviation	Mean volume [mm^3^]					
			Overall	SD	Left	SD	Right	SD
Gray matter	Anterior hypothalamic area	AHA	31.2	4.56	32.71	4.81	29.68	4.45
	Arcuate nucleus	AN	27.34	4.07	28.12	4.15	26.54	4.1
	Bed nucleus of the stria terminalis	BNST	112.86	15.63	117.64	16.4	108.07	15.392
	Dorsal periventricular nucleus	DP	2.87	0.53	2.59	0.48	3.14	0.58
	Dorsomedial hypothalamic nucleus	DM	11.84	1.63	10.32	1.44	13.35	1.88
	Lateral hypothalamus	LH	171.53	22.29	184.66	24.085	158.39	20.93
	Mammillary bodies	MM	73.06	11.46	69.78	10.94	76.33	12.17
	Medial preoptic nucleus	MPO	8.84	1.29	9.31	1.36	8.36	1.24
	Nucleus basalis of Meynert	NBM	50.81	8.18	47.81	7.87	53.79	8.77
	Paraventricular nucleus	PA	39.53	5.7	43.81	6.26	35.24	5.22
	Periventricular nucleus	PE	19.52	2.96	19.83	2.99	19.2	3
	Posterior hypothalamus	PH	25.93	3.35	24.41	3.21	27.43	3.61
	Red nucleus	RN	226.88	33.68	222.66	33.46	231.08	34.67
	Substantia nigra	SN	682.29	91.86	716.69	96.71	647.89	88.37
	Subthalamic nucleus	STN	98.93	14.67	94.08	14.19	103.76	15.5
	Suprachiasmatic nucleus	SCh	4.94	0.86	5.13	0.93	4.74	0.88
	Supraoptic nucleus	SO	16.46	2.37	15.79	2.41	17.13	2.53
	Tuberomammillary nucleus	TM	24.76	3.56	24.34	3.51	25.18	3.72
	Ventromedial hypothalamus	VM	31.7	4.64	28.45	4.2	34.95	5.24
	Zona incerta	ZI	74.14	11.11	70.06	10.8	78.22	11.72
White matter	Anterior commissure	ac	incomplete segmentation					
	Diagonal band of broca	dB	33.42	4.59	39.69	5.64	27.14	3.67
	Fornix	fx	incomplete segmentation					
	Inferior thalamic peduncle	ithp	incomplete segmentation					
	Mammillothalamic tract	mt	incomplete segmentation					

Volumes of gray and white matter structures were estimated following registration of the final atlas segmentations to the 990 transforms obtained from the HCP S1200 subject release. Volumes of incomplete segmentations are not reported. For an itemized description of left and right hemispheric volumes normalized by hemispheric brain volume refer to Supplementary Table 4.

**Table 3:** Estimated absolute volumes of the hypothalamus proper, its major divisions and individual nuclei.

	Preoptic region		Anterior region		Tuberal region		Mammillary region		Mean ± SD [mm^3^]
	Nucleus	Mean ± SD [mm^3^]	Nucleus	Mean ± SD [mm^3^]	Nucleus	Mean ± SD [mm^3^]	Nucleus	Mean ± SD [mm^3^]	
Periventricular zone			Suprachiasmatic nucleus (SC)	4.94 ± 0.86	Arcuate nucleus (AN)	27.34 ± 4.07			94.20 ± 13.40
			**Paraventricular nucleus (PA)**	**39.53 ± 5.70**					
			**Periventricular nucleus (PE)**	19.52 ± 2.96					
Medial zone	Medial preoptic nucleus (MPO)	8.84 ± 1.29	**Paraventricular (PA)**	**39.53 ± 5.70**	Ventromedial nucleus (VM)	31.70 ± 4.64	Posterior hypothalamus (PH)	25.93 ± 3.35	196.20 ± 26.54
			**Periventricular nucleus (PE)**	**19.52 ± 2.96**					
			Anterior hypothalamic area (AHA)	31.20 ± 4.56	Dorsomedial nucleus (DM)	11.84 ± 1.63	**Tuberomammillary nucleus (TM)**	**24.76 ± 3.56**	
Lateral zone			Supraoptic nucleus (SO)	16.46 ± 2.37	Lateral hypothalamus (LH)	171.53 ± 22.29	**Tuberomammillary nucleus (TM)**	**24.76 ± 3.56**	212.10 ± 27.50
Mean ± SD		8.84 ± 1.29		114.52 ± 16.05		242.40 ± 30.88		50.69 ± 6.57	832.90 ± 107.59

Based on its anteroposterior extent, the hypothalamus can be subdivided into preoptic, anterior, tuberal and mammillary region. In contrast, the mediolateral extent is divided into a periventricular, medial and lateral zone. Bold areas indicate duplicates that are associated with two or more subdivisions.

The hypothalamic volumes reported here are within the range of previously published MRI volumetric studies^8,10,22,44^. Previously reported volumes, however, feature a high degree of variability ranging from 600 mm^3^ to 1100 mm^3^. This is in large part owing to the lack of established guidelines for the delineation of the hypothalamus. Furthermore, the structures that were incorporated into the hypothalamus or defined as “hypothalamic” varied considerably among groups. We adopted Mai et al.’s nomenclature to define hypothalamic and extrahypothalamic structures in the present study. To maximize accuracy, hypothalamic nuclei were further segmented by first delineating the anatomical landmarks surrounding the hypothalamus. These structures were readily identifiable (Fig. 2) and facilitated the identification of nuclei within the hypothalamus proper.

### Functional description of hypothalamic nuclei

Functionally, the hypothalamus comprises a myriad of regulatory circuits devoted to the control of basic life functions^5^. The emerging functional networks are remarkably robust, redundant, and complex involving an abundance of distinct cell populations and neurotransmitters^49^. In general, hypothalamic nuclei are implicated in the regulation of circadian rhythm, food intake, thirst, stress response, sexual and defensive behaviors and, thermoregulation^2,50^. Table 4 features an overview of hypothalamic functions and the principal network nodes understood to underlie each function. Supplementary table 1 provides a detailed description of the functional anatomy of each nucleus.

**Table 4:** Hypothalamic involvement in the regulation of circadian, metabolic and autonomic responses.

	AHA	AN	DP	DM	LH	MPO	PA	PE	PH	SCh	SO	TM	VM
Circadian rhythm				O		O	O			X			
Sleep-wake cycle					X							X	
Stress response							X						
Thermoregulation	X					X			X				
Food intake		X		X	X		X						
Thirst		O		O			X				X		
Sexual behavior			O			X		O		O			X
Defensive behavior	X				X								X

Hypothalamic nuclei are tightly interconnected forming a functional network consisting of primary (X) and secondary (O) nodal points. Primary nodal points play a major role in the regulation of the associated function, whereas secondary nodal are known to be implicated in the respective regulatory circuit. AHA, anterior hypothalamic area; AN, arcuate nucleus; DP, dorsal periventricular nucleus; DM, dorsomedial hypothalamic nucleus; LH, lateral hypothalamus; MPO, medial preoptic nucleus; PA, paraventricular nucleus; PE, periventricular nucleus; PH, posterior hypothalamus; SCh, suprachiasmatic nucleus; SO, supraoptic nucleus; TM, tuberomammillary nucleus; VM, ventromedial nucleus. Adopted from Nieuwenhuys R, Voogd J, Huijzen C (2008): The Human Central Nervous System. A Synopsis and Atlas, 4^th^ ed. Springer: Berlin Heidelberg.

### Limitations

There were a few limitations involved with this study. First, segmentation of hypothalamic nuclei was based on standard T1w (3D MPRAGE) and T2w (3D T2-SPACE) data that was obtained from the HCP S1200 subject release. A fuller range of modalities as employed in other segmentation studies may have been useful to better inform delineation of hypothalamic nuclei^11^. Prior investigations, however, were made at the cost of sample size (the study population in Baroncini et al., 2012 consisted of a total of 20 healthy volunteers) and thus lack generalizability. Second, bordering regions of low tissue contrast occasionally lacked clear anatomical boundaries and therefore required raters to make subjective decisions (for a detailed description of the employed segmentation strategy see Supplementary table 1). The employment of two established atlases, as well as minimum deformation averaging of our dataset across 990 patients however, facilitated the process. Finally, relevant white matter bundles, such as mfb, fields of Forel, ansa lenticularis and fasciculus lenticularis that are of clinical interest and known to be located in close proximity to the hypothalamus could not be delineated on structural MRI as they lacked sufficient contrast. Furthermore, as hypothalamic nuclei may be confluent with white matter tracts (e.g. mfb and LH), observed stimulation effects may be the result of remote stimulation of white matter and not directly attributable to direct stimulation of the respective nuclei.

While providing a deeper understanding of anatomical relationships in MNI space, a direct translation of the herein presented hypothalamic atlas into patient space may be detrimental as accurate transformations from MNI space into native patient space cannot be guaranteed. This holds especially true with respect to interindividual neuroanatomical variabilities and morphological changes in pathological conditions^10,44,51^, that cannot be accounted for with normative data from neurotypical young adult brains. Hence, the atlas may be of sufficient detail for clinical applications (i.e. postoperative DBS lead localization, stimulation programming, or evaluation of tumor margins) and research purposes (e.g. volumetric comparisons between healthy and diseased states). However, the authors do not recommend the employment of the atlas as a tool for direct surgical targeting.

## Supplementary information

Supplementary Table 1

Supplementary Table 2

Supplementary Table 3

Supplementary Table 4

Supplementary Table 5

## References

[1] Nieuwenhuys, R., Voogd, J. & Huijzen, C. van. *The Human Central Nervous System. A Synopsis and Atlas* 4th edn (Springer Berlin Heidelberg, 2008).

[2] Saper, C. B. In *The human nervous system* (eds. Mai, J. K. & Paxinos, G.) Ch. 16 (Elsevier Academic Press, 2012).

[3] Barbosa DAN (2017). The hypothalamus at the crossroads of psychopathology and neurosurgery. Neurosurg. Focus.

[4] Le Tissier P (2017). An updated view of hypothalamic-vascular-pituitary unit function and plasticity. Nat. Rev. Endocrinol..

[5] Nieuwenhuys, R., Voogd, J. & Huijzen, C. van. In *The Human Central Nervous System. A synopsis and Atlas* (eds. Nieuwenhuys, R., Voogd, J. & Huijzen, C. van) Ch. 9 (Springer, 2008).

[6] Lemaire JJ (2011). White matter connectivity of human hypothalamus. Brain Res..

[7] Lemaire JJ (2013). Maps of the adult human hypothalamus. Surg. Neurol. Int..

[8] Makris N (2013). Volumetric parcellation methodology of the human hypothalamus in neuroimaging: Normative data and sex differences. Neuroimage.

[9] Bocchetta M (2015). Detailed volumetric analysis of the hypothalamus in behavioral variant frontotemporal dementia. J. Neurol..

[10] Gabery, S. *et al*. Volumetric analysis of the hypothalamus in huntington disease using 3T MRI: The IMAGE-HD study. *PLoS One* **10**, (2015). 10.1371/journal.pone.0117593 PMC4319930 25659157

[11] Baroncini M (2012). MRI atlas of the human hypothalamus. Neuroimage.

[12] Sano K, Mayanagi Y, Sekino H, Ogashiwa M, Ishijima B (1970). Results of stimulation and destruction of the posterior hypothalamus in man. J. Neurosurg..

[13] Torres, C. *et al*. Deep Brain Stimulation for Aggressiveness: Long-Term Follow-Up and Tractography Study of the Stimulated Brain Areas. *J. Neurosurg*. 1–10 (2020). 10.3171/2019.11.JNS192608 32032944

[14] Franzini A, Messina G, Cordella R, Marras C, Broggi G (2010). Deep brain stimulation of the posteromedial hypothalamus: indications, long-term results, and neurophysiological considerations. Neurosurg. Focus.

[15] Franco RR (2018). Assessment of Safety and Outcome of Lateral Hypothalamic Deep Brain Stimulation for Obesity in a Small Series of Patients With Prader-Willi Syndrome. JAMA Netw. open.

[16] Behrens TEJ (2003). Non-invasive mapping of connections between human thalamus and cortex using diffusion imaging. Nat. Neurosci..

[17] Krauth A (2010). A mean three-dimensional atlas of the human thalamus: Generation from multiple histological data. Neuroimage.

[18] Akram H (2018). Connectivity derived thalamic segmentation in deep brain stimulation for tremor. NeuroImage Clin..

[19] Ewert S (2018). Toward defining deep brain stimulation targets in MNI space: A subcortical atlas based on multimodal MRI, histology and structural connectivity. Neuroimage.

[20] Chakravarty MM, Bertrand G, Hodge CP, Sadikot AF, Collins DL (2006). The creation of a brain atlas for image guided neurosurgery using serial histological data. Neuroimage.

[21] Horn A, Blankenburg F (2016). Toward a standardized structural-functional group connectome in MNI space. Neuroimage.

[22] Schindler, S. *et al*. Development and Evaluation of an Algorithm for the Computer-Assisted Segmentation of the Human Hypothalamus on 7-Tesla Magnetic Resonance Images. *PLoS One* **8**, (2013). 10.1371/journal.pone.0066394 PMC3720799 23935821

[23] Pauli WM, Nili AN, Tyszka JM (2018). A high-resolution probabilistic *in vivo* atlas of human subcortical brain nuclei. Sci. Data.

[24] Glasser MF (2013). The minimal preprocessing pipelines for the Human Connectome Project. Neuroimage.

[25] Janke AL, Ullmann JFP (2015). Robust methods to create *ex vivo* minimum deformation atlases for brain mapping. Methods.

[26] Sadedin SP, Pope B, Oshlack A (2012). Bpipe: A tool for running and managing bioinformatics pipelines. Bioinformatics.

[27] Avants, B. B., Tustison, N. & Song, G. Advanced Normalization Tools (ANTS). *Insight J*. 1–35 (2014).

[28] Eskildsen SF (2012). BEaST: Brain extraction based on nonlocal segmentation technique. Neuroimage.

[29] Mazziotta JC, Toga AW, Evans A, Fox P, Lancaster J (1995). A probabilistic atlas of the human brain: theory and rationale for its development. The International Consortium for Brain Mapping (ICBM). Neuroimage.

[30] Mai, J. K., Paxinos, G. & Voss, T. *Atlas of the Human Brain*. 4th edn (Elsevier Academic Press, 2016).

[31] Hawrylycz MJ (2012). An anatomically comprehensive atlas of the adult human brain transcriptome. Nature.

[32] Schlaepfer TE, Bewernick BH, Kayser S, Mädler B, Coenen VA (2013). Rapid effects of deep brain stimulation for treatment-resistant major depression. Biol. Psychiatry.

[33] Coenen VA (2018). Tractography-assisted deep brain stimulation of the superolateral branch of the medial forebrain bundle (slMFB DBS) in major depression. NeuroImage Clin..

[34] Pipitone J (2014). Multi-atlas segmentation of the whole hippocampus and subfields using multiple automatically generated templates. Neuroimage.

[35] Carass A (2020). Evaluating White Matter Lesion Segmentations with Refined Sørensen-Dice Analysis. Sci. Rep..

[36] Neudorfer C (2020). Zenodo.

[37] Dice LR (1945). Measures of the Amount of Ecologic Association Between Species. Ecology.

[38] Rockafellar, R. T. & Wets, R. J.-B. *Variational Analysis*. 3rd edn (Springer Berlin Heidelberg, 2009).

[39] Deeb W (2019). Fornix-Region Deep Brain Stimulation–Induced Memory Flashbacks in Alzheimer’s Disease. N. Engl. J. Med..

[40] Horn A (2019). Lead-DBS v2: Towards a comprehensive pipeline for deep brain stimulation imaging. Neuroimage.

[41] Koutcherov Y, Mai JK, Ashwell KW, Paxinos G (2004). Organisation of the human dorsomedial hypothalamic nucleus. Neuroreport.

[42] Rabin BM (1974). Independence of food intake and obesity following ventromedial hypothalamic lesions in the rat. Physiol. Behav..

[43] Parkinson WL, Weingarten HP (2017). Dissociative analysis of ventromedial hypothalamic obesity syndrome. Am. J. Physiol. Integr. Comp. Physiol..

[44] Goldstein JM (2007). Hypothalamic Abnormalities in Schizophrenia: Sex Effects and Genetic Vulnerability. Biol. Psychiatry.

[45] Callen DJA, Black SE, Gao F, Caldwell CB, Szalai JP (2001). Beyond the hippocampus: MRI volumetry confirms widespread limbic atrophy in AD. Neurology.

[46] Klomp A, Koolschijn PCMP, Hulshoff Pol HE, Kahn RS, Van Haren NEM (2012). Hypothalamus and pituitary volume in schizophrenia: A structural MRI study. Int. J. Neuropsychopharmacol..

[47] Peper JS (2010). HPG-axis hormones during puberty: A study on the association with hypothalamic and pituitary volumes. Psychoneuroendocrinology.

[48] Piguet O (2011). Eating and hypothalamus changes in behavioral-variant frontotemporal dementia. Ann. Neurol..

[49] Hahn JD, Sporns O, Watts AG, Swanson LW (2019). Macroscale intrinsic network architecture of the hypothalamus. Proc. Natl. Acad. Sci. USA.

[50] Saper CB, Lowell BB (2014). The hypothalamus. Curr. Biol..

[51] Breen DP (2016). Hypothalamic volume loss is associated with reduced melatonin output in Parkinson’s disease. Mov. Disord..

