# Supplementary material for: A high-resolution in vivo magnetic resonance imaging atlas of the human hypothalamic region
Source: Sci Data. 2020 Sep 15;7:305. doi: 10.1038/s41597-020-00644-6 (PMC7492465; doi:10.1038/s41597-020-00644-6)
Supplement: Supplementary file 3 — Supplementary Table 3 [file 41597_2020_644_MOESM3_ESM.pdf]

SUPPLEMENTARY TABLE 3

Nucleus	Function	Delineation
**Anterior hypothalamic area (AHA)**	The nucleus constitutes the rostral heat control center and responds to increases in blood and skin temperature[52,53]. During electrical stimulation cutaneous vasodilation and sweating can be observed[54]. Damage to AHA may yield diverse thermoregulatory changes including hyperthermia, hypothermia and poikilothermia[55].	According to the atlas of the human brain, AHA constitutes a diffuse accumulation of cells on coronal brain sections. Similarly, the nucleus presented in isosignal on T1w and T2w images and was thus not easily identifiable. Because of this circumstance, the nucleus was only defined at a late stage in the segmentation process after the boundaries to its adjacent nuclei had been established.
**Arcuate nucleus (AN)**	AN is the major site of leptin receptor expression in the hypothalamus and contains neurons relevant for the control of growth and feeding behavior. Furthermore, the nucleus is sensitive to growth-hormone releasing hormone (GHRH) and promotes processes associated with growth, cell reproduction and cell regeneration[56,57]. Structurally, AN can be subdivided into a lateral aspect which belongs to the melanocortin system and a medial aspect that contains agouti-related protein (AgRP), GABA and neuropeptide Y (NPY), respectively. Leptin that circulates in the bloodstream exerts inhibitory effects on AgRP releasing neurons while promoting the release of proopiomelanocortin (POMC) in the lateral portion. POMC acts upon the paraventricular nucleus (Pa) eventually reducing food intake and increasing energy expenditure[58,59]. Conversely, low leptin levels disinhibit AgRP neurons in the medial aspect and promote food intake via Pa[60].	Owing to its close proximity to the third ventricle and the interpeduncular cistern, AN was clearly delimitable on T1w and T2w sequences. The ventral border of AN and its demarcation from VM and Pe proved difficult and was primarily based on qualitative morphological analysis using the Atlas of the Human Brain[61].
**Dorsomedial hypothalamic nucleus (DM)**	The nucleus constitutes a neural relay in the central control of homeostasis in humans. Studies have implicated DM in the regulation of metabolism and food intake that is mediated by a variety of appetite regulating molecules such as leptin, orexin/hypocretin, neuropeptide Y and neurotensin[62–64]. It further relays information to brain regions associated with the regulation of stress, temperature and cardiovascular function[65].	Identification of DM proved difficult on both T1w and T2w sections, which is likely attributable to the loosely dispersed neurons whose morphology resembles the surrounding hypothalamic gray matter[65]. Overall, the T1w sequence proved more useful in the distinction of DM.
**Lateral hypothalamus (LH)**	LH is the primary orexinergic center within the hypothalamus and mediates an array of functions through diverse outputs. Two of its most commonly noted functions include the promotion of food intake and arousal (i.e. wakefulness), which has led to the investigation of the nucleus in maladaptive feeding behavior, namely obesity and anorexia. The relevance of orexins in feeding behavior were first recognized based on the finding that exogenous application of orexins (*orexis* means “appetite”) increased food intake[66], while blockage of the orexin receptor was associated with anorectic effects[67]. Similarly, electrocoagulation of LH in patients with obesity was shown to temporarily suppress appetite and slightly reduce body weight[68]. The role of orexins in appetitive behavior has since been extended to other reward-related behaviors, such as drug abuse and addiction[69].<br><br>Clinical evidence for the role of orexins in the maintenance of wakefulness has been accumulated in patients suffering from narcolepsy, a disease characterized by loss of orexin-producing neurons	On coronal sections, LH is confined medially by both the fornix and the mammillothalamic tract. Its lateral boundary constitutes the medial margin of the internal capsule. These white matter structures feature strong contrast with respect to LH and allowed a clear delineation of the structure on T1w MRI sequences.

	in LH[70,71]. Owing to the absence of LH input, potent projections to the arousal system cannot be engaged in this condition, leading to overwhelming sleep attacks and excessive tiredness[60].<br><br>In addition to orexin, LH is implicated in the synthesis of a variety of neurotransmitters, such as melanin-concentrating hormone (MCH) and GABA that modulate and fine-tune the activity levels of orexin neurons[72].	
**Medial preoptic nucleus (MPO)**	The nucleus is a key integrator of thermal inputs from both the skin and temperature-sensitive neurons within the brain. Exposure to pyrogens (e.g. prostaglandin E2) causes a shift of set-point temperatures in MPO leading to the induction of fever[53]. Furthermore, the nucleus releases gonadotropin-releasing hormone (GnRH) and is implicated in reproductive behavior. In consequence, MPO features a sexual dimorphism that is reflected in gender-specific differences in volume, structure and cell density[73,74]. During sexual stimulation, individual neurons in MPO were shown to be active[60]. Lesions consequently disrupt the autonomic and motor patterns associated with sexual behavior and lead to loss of copulatory behaviors[75].	Located between LH and the paraventricular nucleus, MPO was readily identifiable on T1w sequences.
**Paraventricular nucleus (PA)**	The magnocellular portion of the nucleus synthesizes the neuropeptides vasopressin (antidiuretic hormone, ADH) and oxytocin that are released into the bloodstream via the posterior pituitary. While ADH plays an important role in the regulation of water balance, oxytocin stimulates the contraction of smooth muscle in the mammillary glands and the uterus. The medial parvocellular and periventricular portion comprise corticotrophin-releasing hormone (CRH) and thyrotropin-releasing hormone (TRH) immunoreactive neurons[76,77]. By promoting the release of triiodothyronine (T3) and thyroxin (T4) Pa is involved in the regulation of cardiovascular, metabolic and thermoregulatory processes[78]. CRH plays a crucial role in the response to stress by promoting the release of glucocorticoids (corticosterone and cortisol) from the adrenal gland. Finally, the lateral parvocellular and dorsal areas of Pa feature descending connections to autonomic nuclei of the brain stem and spinal cord[79].	PA could be clearly identified on both T1w and T2w sequences owing to its location immediately beneath the third ventricle and its sharp boundary with the periventricular nucleus.
**Periventricular nucleus (PE)**	Somatostatin released from PE and GHRH released from AN coordinate the episodic secretion of growth hormone (GH) into the bloodstream. On the hypothalamic level, this is achieved via reciprocal connections between GHRH neurons and somatostatin neurons. Hormonal feedback from the periphery (GH and IGF-1) further alters hypothalamic output, contributing to a finely tuned regulatory system[80]. Different levels of somatostatin secretion between males and females are believed to play a role in the sexual dimorphism of growth hormone secretion[81].	Both the ventral and the dorsal border of PE were difficult to delineate on T1w and T2w sections and segmentation was largely based on the relative position to surrounding brain structures as well as the atlas of the human brain. Conversely, the mediolateral expansion of the nucleus could be identified on both T1w and T2w images.
**Posterior hypothalamus (PH)**	Also known as the caudal heat control center. In contrast to AHA, the nucleus is temperature blind i.e. neurons do not respond directly to changes in temperature[82]. Electrical and chemical stimulation, however, were shown to induce vasoconstriction, shivering and increased metabolic heat production[83]. In contrast, lesions within PH have been shown to impair heat production causing poikilohtermia[55]. Radiofrequency (RF) lesioning of the posteromedial part of PH that was shown to be effective in the treatment of aggressive behavior was associated with sympathetic responses suggesting involvement of PH in the processing of autonomic signals[84]. More recently, PH has been	With respect to white matter structures such as the mammillothalamic tract the outline of PH was fairly clear on T1w sequences.

	identified as a potential target in the treatment of drug-resistant chronic cluster headache (CCH)[85–87].	
**Suprachiasmatic nucleus (SCh)**	Pacemaker cells within the suprachiasmatic nucleus (SCh) are responsible for the integration of circadian fluctuations and adjustment of endocrine activity in response[88]. To mitigate these adaptive processes, the nucleus receives direct projections from the retina[89,90]. Output from SCh is then translated into circadian rhythms of sleeping, feeding, thermoregulation, locomotor activity and corticosteroid secretion[91]. While SCh directly promotes wakefulness and sleep via the release of melatonin in the pineal gland, mono- and disynaptic intrahypothalamic projections allow a flexible adjustment of hormone secretion in response to environmental cues such as ambient temperature, food availability and social interactions[92].	The boundaries of SCh were poorly discernable on MRI imaging modalities and its delineation was primarily based on brain atlases and surrounding landmarks such as the optic chiasm and AC.
**Supraoptic nucleus (SO)**	Analogous to PA the SO is involved in the synthesis of ADH and Oxytocin and promotes antidiuresis and smooth muscle contraction of the uterus and mammary glands, respectively. While the majority of neurons within SO is dedicated towards the production of ADH, only 10-15% of cells account for the synthesis of oxytocin[93,94].	As its name indicates, SO rests on top of the optic chiasm extending towards the optic tract. Thus, the nucleus was usually correctly identifiable on T1w sequences.
**Tuberomammillary nucleus (TM)**	TM is considered the sole source of histaminergic neurons within the CNS. Its projections disseminate across the CNS densely innervating the cerebral cortex, striatum, substantia nigra, thalamus, amygdala, hippocampus, cerebellum and spinal cord[95]. TM activity and the extent of histamine release is tightly coupled to the behavioral state. While increased neuronal firing can be observed during attention and waking, neuronal activity subsides or arrests during non-rapid eye movement (NREM) and rapid eye movement (REM) sleep, respectively[96,97].	TM could be identified on T1w images and based on its bordering neuroanatomical structures such as fornix, LH and ventromedial hypothalamus (VM).
**Ventromedial hypothalamus (VM)**	VM is a distinct morphological nucleus that is associated with satiety and has been implicated in the etiology of obesity. The nucleus comprises a large population of glucose-sensitive neurons that dynamically respond to hypoglycemia[98]. Furthermore, it possesses an abundance of receptors sensitive to dopamine, serotonin, GABA, histamine and estrogen that all affect feeding behavior. In obese animals, the functional integrity of these receptors has been found to be abnormal[60]. Lesions of VM are associated with weight gain and hyperphagia. Owing to VM's involvement in the processing of autonomic function, these metabolic changes are also in part attributable to altered sympathetic and parasympathetic responses[99,100].<br><br>In addition to the termination of hunger VM is implicated in fear, sexual activity and thermoregulation. Similar to MPO, the nucleus is sexually dimorphic and mediates motor patterns associated with sexual behavior. In consequence, VM has been employed as a stereotactic target in very controversial and ethically questionable stereotactic lesioning procedures in the treatment of "sexually deviant" behavior[101,102].	The oval shape and the cell-poor zone surrounding VM allowed a direct identification of the nucleus on MRI sections in the coronal plane. The T1w sequence proved particularly useful for this task.

Supplementary Table 1: Functional description of hypothalamic nuclei and labeling criteria used to identify and delineate individual structures. The hypothalamus is involved in the regulation and homeostasis of circadian rhythm, sleep-wake cycle, stress response, thermoregulation, food intake, thirst, sexual behavior, and defensive behavior. Functions are regulated in robust and redundant networks within dedicated nuclei.

References

52. Zhao, Z.-D. *et al.* A hypothalamic circuit that controls body temperature. *Proc. Natl. Acad. Sci. U. S. A.* **114**, 2042–2047 (2017).
53. Boulant, J. A. Role of the Preoptic-Anterior Hypothalamus in Thermoregulation and Fever. *Clin. Infect. Dis.* **31**, S157–S161 (2002).
54. Scammell, T. E., Price, K. J. & Sagar, S. M. Hyperthermia induces c-fos expression in the preoptic area. *Brain Res.* **618**, 303–307 (1993).
55. Swaab, D. F. (Dick F., Aminoff, M. J. (Michael J. & Boller, F. *The human hypothalamus : basic and clinical aspects. Part II, Neuropathology of the human hypothalamus and adjacent brain structures*. (Elsevier, 2004).
56. Bloch, B., Gaillard, R. C., Brazeau, P., Lin, H. D. & Ling, N. Topographical and ontogenetic study of the neurons producing growth hormone-releasing factor in human hypothalamus. *Regul. Pept.* **8**, 21–31 (1984).
57. Pelletier, G., Désy, L., Côté, J., Lefèvre, G. & Vaudry, H. Light-microscopic immunocytochemical localization of growth hormone-releasing factor in the human hypothalamus. *Cell Tissue Res.* **245**, 461–463 (1986).
58. Ahlma, R. S. *et al.* Role of leptin in the neuroendocrine response to fasting. *Nature* **382**, 250–252 (1996).
59. Cheung, C. C., Clifton, D. K. & Steiner, R. A. Proopiomelanocortin neurons are direct targets for leptin in the hypothalamus. *Endocrinology* **138**, 4489–4492 (1997).
60. Saper, C. B. & Lowell, B. B. The hypothalamus. *Curr. Biol.* **24**, R1111–R1116 (2014).
61. Mai, J. K., Paxinos, G. & Voss, T. *Atlas of the Human Brain*. (Academic Press, 2016).
62. Bellinger, L. L. & Bernardis, L. L. The dorsomedial hypothalamic nucleus and its role in ingestive behavior and body weight regulation: Lessons learned from lesioning studies. in *Physiology and Behavior* **76**, 431–442 (2002).
63. Abbott, C. R. *et al.* Identification of hypothalamic nuclei involved in the orexigenic effect of melanin-concentrating hormone. *Endocrinology* **144**, 3943–3949 (2003).
64. Elmquist, J. K., Elias, C. F. & Saper, C. B. From lesions to leptin: Hypothalamic control of food intake and body weight. *Neuron* **22**, 221–232 (1999).
65. Koutcherov, Y., Mai, J. K., Ashwell, K. W. & Paxinos, G. Organisation of the human dorsomedial hypothalamic nucleus. *Neuroreport* **15**, 107–111 (2004).
66. Sakurai, T. *et al.* Orexins and orexin receptors: A family of hypothalamic neuropeptides and G protein-coupled receptors that regulate feeding behavior. *Cell* **92**, 573–585 (1998).
67. Haynes, A. C. *et al.* A selective orexin-1 receptor antagonist reduces food consumption in male and female rats. in *Regulatory Peptides* **96**, 45–51 (2000).
68. Quaade, F. Stereotaxy for obesity. *The Lancet* **303**, 267 (1974).
69. James, M. H., Mahler, S. V, Moorman, D. E. & Aston-Jones, G. A Decade of Orexin/Hypocretin and Addiction: Where Are We Now? *Curr. Top. Behav.*

Neurosci. **33**, 247–281 (2017).

70. Nishino, S., Ripley, B., Overeem, S., Lammers, G. J. & Mignot, E. Hypocretin (orexin) deficiency in human narcolepsy. *Lancet* **355**, 39–40 (2000).
71. Peyron, C. *et al.* A mutation in a case of early onset narcolepsy and a generalized absence of hypocretin peptides in human narcoleptic brains. *Nat. Med.* **6**, 991–997 (2000).
72. Burt, J., Alberto, C. O., Parsons, M. P. & Hirasawa, M. Local network regulation of orexin neurons in the lateral hypothalamus. *Am. J. Physiol. Integr. Comp. Physiol.* **301**, R572–R580 (2011).
73. Swaab, D. F. & Hofman, M. A. Sexual differentiation of the human hypothalamus in relation to gender and sexual orientation. *Trends in Neurosciences* **18**, 264–270 (1995).
74. Ferretti, A. *et al.* Dynamics of male sexual arousal: Distinct components of brain activation revealed by fMRI. *Neuroimage* **26**, 1086–1096 (2005).
75. Numan, M. Sexual Behaviors and Sexual Differentiation. in *Neurobiology of Social Behavior* 109–164 (2015). doi:10.1016/b978-0-12-416040-8.00004-3
76. Fliers, E., Noppen, N. W. A. M., Wiersinga, W. M., Visser, T. J. & Swaab, D. F. Distribution of thyrotropin-releasing hormone (TRH)-containing cells and fibers in the human hypothalamus. *J. Comp. Neurol.* **350**, 311–323 (1994).
77. Pelletier, G., Désy, L., Côté, J. & Vaudry, H. Immunocytochemical localization of corticotropin-releasing factor-like immunoreactivity in the human hypothalamus. *Neurosci. Lett.* **41**, 259–263 (1983).
78. Mullur, R., Liu, Y.-Y. & Brent, G. A. Thyroid Hormone Regulation of Metabolism. *Physiol. Rev.* **94**, 355–382 (2014).
79. Ad, L. Forebrain nuclei involved in autonomic control. *Prog. Brain Res.* **87**, 253–268 (1990).
80. Epelbaum, J. Intrahypothalamic Neurohormonal Interactions in the Control of Growth Hormone Secretion. in 54–68 (2007). doi:10.1002/9780470514283.ch5
81. Murray, H. E., Simonian, S. X., Herbison, A. E. & Gillies, G. E. Correlation of hypothalamic somatostatin mRNA expression and peptide content with secretion: Sexual dimorphism and differential regulation by gonadal factors. *J. Neuroendocrinol.* **11**, 27–33 (1999).
82. Nieuwenhuys, R., Voogd, J. & Huijzen, C. van. Telencephalon: Amygdala and Claustrum. in *The Human Central Nervous System. A synopsis and Atlas* 967 (2008). doi:10.1007/978-3-540-34686-9
83. Thornhill, J. A. & Halvorson, I. Electrical stimulation of the posterior and ventromedial hypothalamic nuclei causes specific activation of shivering and nonshivering thermogenesis. *Can. J. Physiol. Pharmacol.* **72**, 89–96 (2011).
84. Rizzi, M. *et al.* Exploring the brain through posterior hypothalamus surgery for aggressive behavior. *Neurosurg. Focus* **43**, E14 (2017).
85. Franzini, A. *et al.* Stimulation of the posterior hypothalamus for treatment of chronic intractable cluster headaches: First reported series. *Neurosurgery* **52**, 1095–1101 (2003).

86. Schoenen, J. *et al.* Hypothalamic stimulation in chronic cluster headache: A pilot study of efficacy and mode of action. *Brain* **128**, 940–947 (2005).
87. Leone, M., Franzini, A., Broggi, G. & Bussone, G. Hypothalamic stimulation for intractable cluster headache: Long-term experience. *Neurology* **67**, 150–152 (2006).
88. Swaab, D. F., Fliers, E. & Partiman, T. S. The suprachiasmatic nucleus of the human brain in relation to sex, age and senile dementia. *Brain Res.* **342**, 37–44 (1985).
89. Sadun, A. A., Schaechter, J. D. & Smith, L. E. H. A retinohypothalamic pathway in man: Light mediation of circadian rhythms. *Brain Res.* **302**, 371–377 (1984).
90. Dai, J., Van Der Vliet, J., Swaab, D. F. & Buijs, R. M. Human retinohypothalamic tract as revealed by in vitro postmortem tracing. *J. Comp. Neurol.* **397**, 357–370 (1998).
91. Moore, R. Y. Circadian rhythms: basic neurobiology and clinical applications. *Annu. Rev. Med.* **48**, 253–66 (1997).
92. Saper, C. B., Lu, J., Chou, T. C. & Gooley, J. The hypothalamic integrator for circadian rhythms. *Trends in Neurosciences* **28**, 152–157 (2005).
93. Van der Woude, P. F. *et al.* No vasopressin cell loss in the human hypothalamus in aging and Alzheimer's disease. *Neurobiol. Aging* **16**, 11–8
94. Wierda, M. *et al.* Oxytocin cell number in the human paraventricular nucleus remains constant with aging and in Alzheimer's disease. *Neurobiol. Aging* **12**, 511–516 (1991).
95. Haas, H. & Panula, P. The role of histamine and the tuberomamillary nucleus in the nervous system. *Nat. Rev. Neurosci.* **4**, 121–130 (2003).
96. Saper, C. B., Chou, T. C. & Scammell, T. E. The sleep switch: Hypothalamic control of sleep and wakefulness. *Trends in Neurosciences* **24**, 726–731 (2001).
97. Ko, E. M., Estabrooke, I. V., McCarthy, M. & Scammell, T. E. Wake-related activity of tuberomammillary neurons in rats. *Brain Res.* **992**, 220–226 (2003).
98. King, B. M. The rise, fall, and resurrection of the ventromedial hypothalamus in the regulation of feeding behavior and body weight. *Physiology and Behavior* **87**, 221–244 (2006).
99. Rabin, B. M. Independence of food intake and obesity following ventromedial hypothalamic lesions in the rat. *Physiol. Behav.* **13**, 769–772 (1974).
100. Parkinson, W. L. & Weingarten, H. P. Dissociative analysis of ventromedial hypothalamic obesity syndrome. *Am. J. Physiol. Integr. Comp. Physiol.* **259**, R829–R835 (2017).
101. Roeder, F. & Müller, D. The stereotaxic treatment of paedophilic homsexuality. *Ger. Med. Mon.* **14**, 265–271 (1969).
102. Müller, D., Roeder, F. & Orthner, H. Further Results of Stereotaxis in the Human Hypothalamus in Sexual Deviations: First Use of this Operation in Addiction to Drugs. *Neurochirurgica* **16**, 113–126 (1973).
